# An optimized integrin α6‐targeted peptide for positron emission tomography/magnetic resonance imaging of pancreatic cancer and its precancerous lesion

**DOI:** 10.1002/ctm2.157

**Published:** 2020-08-26

**Authors:** Yan Mei, Ying‐He Li, Xiao‐Chun Yang, Chao Zhou, Zhi‐Jian Li, Xiao‐Bin Zheng, Jia‐Cong Ye, Cheng Li, Xuan‐Hong Zhang, Jian‐Min Yuan, Hui‐Qiang Huang, Wei Fan, Wei‐Guang Zhang, Mu‐Sheng Zeng, Guo‐Kai Feng

**Affiliations:** ^1^ State Key Laboratory of Oncology in South China Collaborative Innovation Center for Cancer Medicine Sun Yat‐sen University Cancer Center Guangzhou China; ^2^ School of Pharmaceutical Sciences Sun Yat‐sen University Guangzhou China; ^3^ Experimental Equipment Management Center, Zhongshan School of Medicine Sun Yat‐Sen University Guangzhou China; ^4^ Central Research Institute UIH Group Shanghai China

Dear Editor,

Recently, we successfully developed a radiolabeled probe targeting integrin α6 for the positron emission tomography (PET)/magnetic resonance (MR) imaging of pancreatic ductal adenocarcinoma (PDAC) and its precancerous lesion, pancreatic intraepithelial neoplasia (PanIN).

Most pancreatic cancers are PDAC.^1^ Standard approaches for detecting PDAC at early stage are lacking with most diagnosed patients at advanced stage.^2^ Tumor‐targeted molecular imaging significantly improved the tumor‐detection rate,^3^ which may also enhance the early diagnosis of PDAC. Considering the genetic complexity of PDAC, a PDAC‐specific target with highly adaptability, sensitivity, and specificity is required.

We selected integrin α6 as the target of PDAC as it was overexpressed in pancreatic cancer cells^4^, ^5^ and cancerous tissue of patients with PDAC^4^, ^6^, ^7^ with an overexpression rate up to 92%.^4^ Increased integrin α6 levels were associated with stronger PDAC cell invasiveness^5^ and poorer prognosis for patients.^6^, ^7^ Furthermore, the integrin α6β4 dimer was overexpressed in PanIN even at a stage as early as PanIN 1A.^4^ Here, we confirmed that integrin α6 was overexpressed in human PDAC (Figure 1A,B) and increased integrin α6 had poorer prognosis for PDAC patients (Figure 1C). Recently, a peptide CRWYDENAC (dubbed RWY) targeting integrin α6 has been identified^8^ and employed for nanotherapeutics of nasopharyngeal carcinoma^8^ and for PET imaging of hepatocellular carcinoma by our team.^9^ Here, we used the Ala scanning mutagenesis to modify the RWY peptide. R, W, and Y were the three key amino acids within the RWY peptide, since their replacement by an alanine reduced the cellular binding ability of this peptide, both in vitro and in vivo. Interestingly, the alanine substitution of E resulted in the CRWYDANAC (dubbed S5) peptide with an approximately 1.5‐fold enhanced tumor binding ability (Figure 1D,E). The higher binding affinity of S5 peptide to tumor cells was further confirmed in vitro by flow cytometry (Figure 1F,G) and in vivo in PDAC mouse model using optical imaging (Figure 1H,I).

**FIGURE 1:**
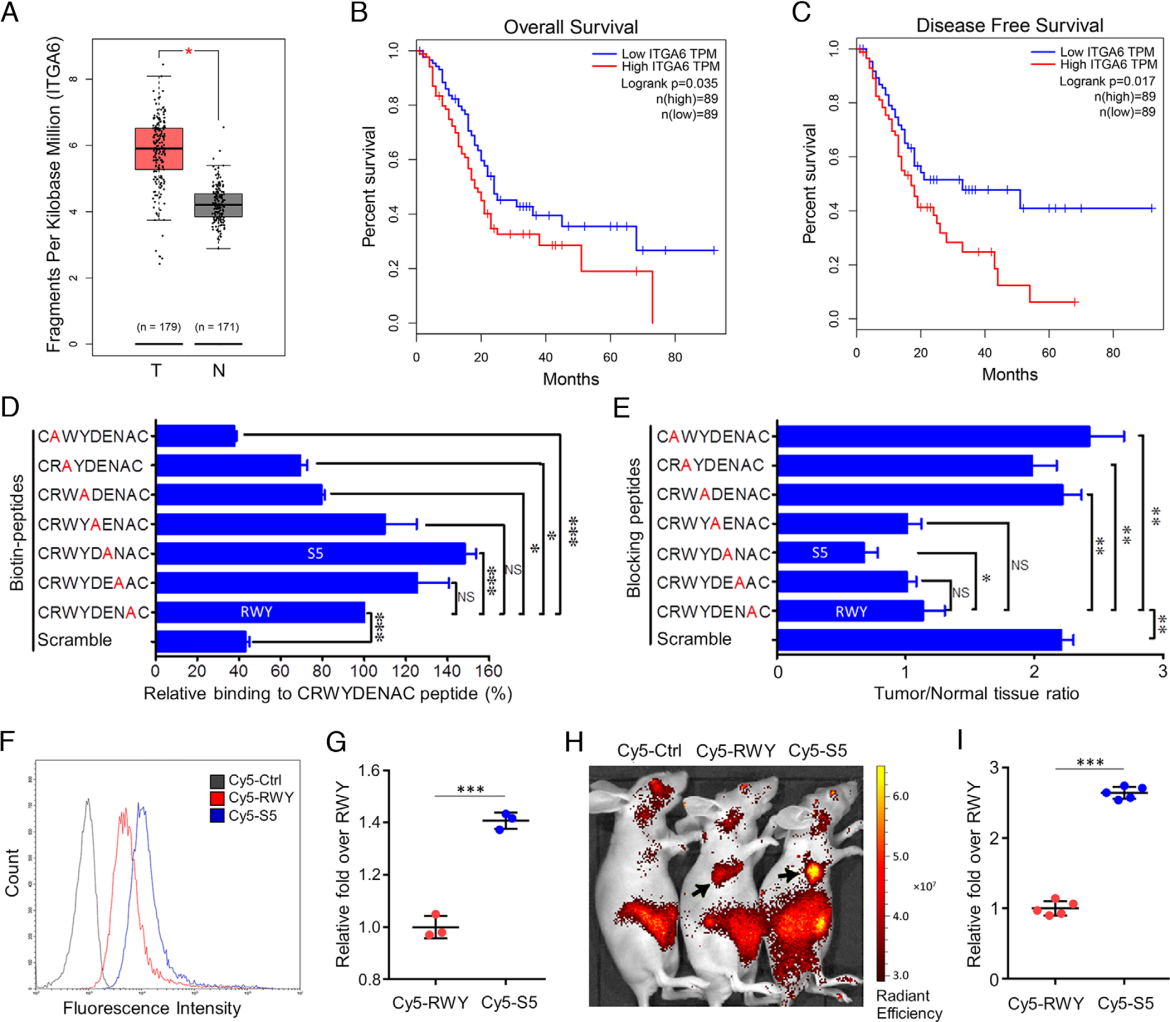
Increased and poorer prognosis of integrin α6 in PDAC and the optimization of a peptide targeting integrin α6. A, According to the TCGA database, increased integrin α6 expression was observed in tumor tissues of PADC patients. B and C, Based on the GEPIA database, patients with higher integrin α6 expression in the tumor tissue showed a poorer prognosis for overall and disease‐free survival. D, The binding affinities of seven Ala‐mutated peptides were analyzed by ELISA. E, The in vivo blocking assays were performed via intravenous injection of 6 nmol of Cy5‐RWY together with 6 μmol of the unlabeled RWY peptide, or its scrambled peptide or a set of its alanine‐scanning mutant peptides into mice bearing Sw1990 tumors. The S5 peptide had the highest blocking effect on Cy5‐RWY. F and G, Sw1990 cells were incubated with Cy5‐RWY and Cy5‐S5 at 37°C for 30 min, and the integrin α6‐binding affinities of Cy5‐RWY and Cy5‐S5 were analyzed by flow cytometric analysis (F); the fluorescence intensity of the S5 group presented as relative (1.41 ± 0.03) fold over RWY, indicating a higher tumor cell‐binding ability of S5. H and I To further investigate the in vivo tumor‐binding ability of the S5 peptide, Cy5‐S5, Cy5‐RWY, and Cy5‐CG7C (negative control) were synthesized and used in NIRF. In the in vivo NIRF imaging of mice bearing subcutaneous Sw1990‐luciferase xenografts, Cy5‐S5 exhibited higher fluorescence intensity in tumor location (black arrow: the xenograft) (H); the fluorescence intensity of Cy5‐S5 presented as relative fold over RWY (2.64 ± 0.09) was significantly higher than that of Cy5‐RWY (1.00 ± 0.10, *P* = .000) (mean ± SD, ANOVA in (D) and (E) and Student's *t*‐test in (G) and (I) were used for statistical analysis. *P < .05, **P < .01, ***P < .001. NS, not significant)

The recently introduced PET/MR imaging technique displays certain advantages in tumor imaging and has potential applications in the imaging of early pancreatic cancer.^10^ Thus, we synthesized the radiotracer ^18^F‐NOTA‐PEG4‐S5 (^18^F‐S5 for short) for the PET/MR imaging (Figure S1A). ^18^F‐S5 was stable in serum (Figure S1B), and it showed ideal biodistribution characteristics in vivo that the uptake of ^18^F‐S5 in tumor tissues was the highest among all collected tissues and organs except for the kidney (Figure S1C,D, Table S1). The convenience of ^18^F‐S5‐PET/MR imaging on PDAC and PanIN was proved in four mice models. Approximately 3.7 × 10^6^ Bq (100 μCi) of ^18^F‐S5 was intravenously injected to a mouse for PET imaging, and 2.5 mmol/kg of gadolinium‐diethylenetriamine pentaacetic acid was injected for enhanced MR imaging. After 120 min postinjection of ^18^F‐S5, PET/MR imaging was obtained by using a hybrid 3.0T PET/MR scanner (uPMR 790; UIH, Shanghai, China).

The PDAC‐targeting effect of ^18^F‐S5 was preliminary examined in a subcutaneous Sw1990‐luciferase tumor‐bearing mouse model, where the tumor‐to‐muscle ratio reached 3.62 ± 0.25 in unblocked mice and significantly decreased to 1.62 ± 0.05 when the binding was blocked by nonradiolabeled S5 (Figure S2A‐D). Similar results were observed in the orthotopic Sw1990‐luciferase mouse model with a tumor‐to‐muscle ratio of 3.48 ± 0.62 (Figure S2E‐G). In the genetically engineered KPC mice (LSL‐Kras^G12D/+^; Pdx1‐Cre; Trp53^fl/fl^) with spontaneously developed PDAC, a routine MR scan showed no abnormal signal in pancreas on T1 weighted images, and the tumor only presented as local moderate enhancement on the enhanced MR scans (Figure S3A). However, in the PET/MR imaging, ^18^F‐S5 produced high PET signals in the possible location of the tumor (Figure 2A). The uptake of ^18^F‐S5 in tumor was quantified as 4.34 ± 1.16 %ID/g (a percentage of the injected dose per gram) (n = 5) and the tumor‐to‐muscle ratio was 4.32 ± 0.65 (Figure 2B). The pancreas of the KPC mouse displayed morphological changes compared with the pancreas of a healthy mouse (Figure 2C). The overexpression of integrin α6 in the spontaneously developed PDAC tissue was confirmed using immunohistochemistry (Figure 2D). In addition, ^18^F‐S5‐PET/MR imaging could also detect metastatic tumors in KPC, where ^18^F‐S5 produced high PET signals in both of the PDAC lesions and the metastatic tumors in the abdominal cavity (Figure S4A‐D). Furthermore, ^18^F‐S5‐PET/MR imaging could detect precancerous lesions of PDAC in the KC mice (LSL‐Kras^G12D/+^; Pdx1‐Cre) with spontaneous developed PanIN (Figure 2E), which was undetectable when enhanced MR imaging was used alone (Figure S3B). ^18^F‐S5 showed a high accumulation in the possible lesion site, as the quantified uptake was 2.99 ± 0.52 %ID/g and the PanIN‐to‐muscle ratio was 3.35 ± 0.43 (Figure 2F). The pancreas of the KC mouse displayed morphological changes as its outer surface was rougher (Figure 2G ) than that of the normal pancreas, as shown in Figure 2C. Immunohistochemical analysis confirmed the formation of PanIN lesions in the pancreas and increased integrin α6 expression in lesion tissues (Figure 2H).

**FIGURE 2:**
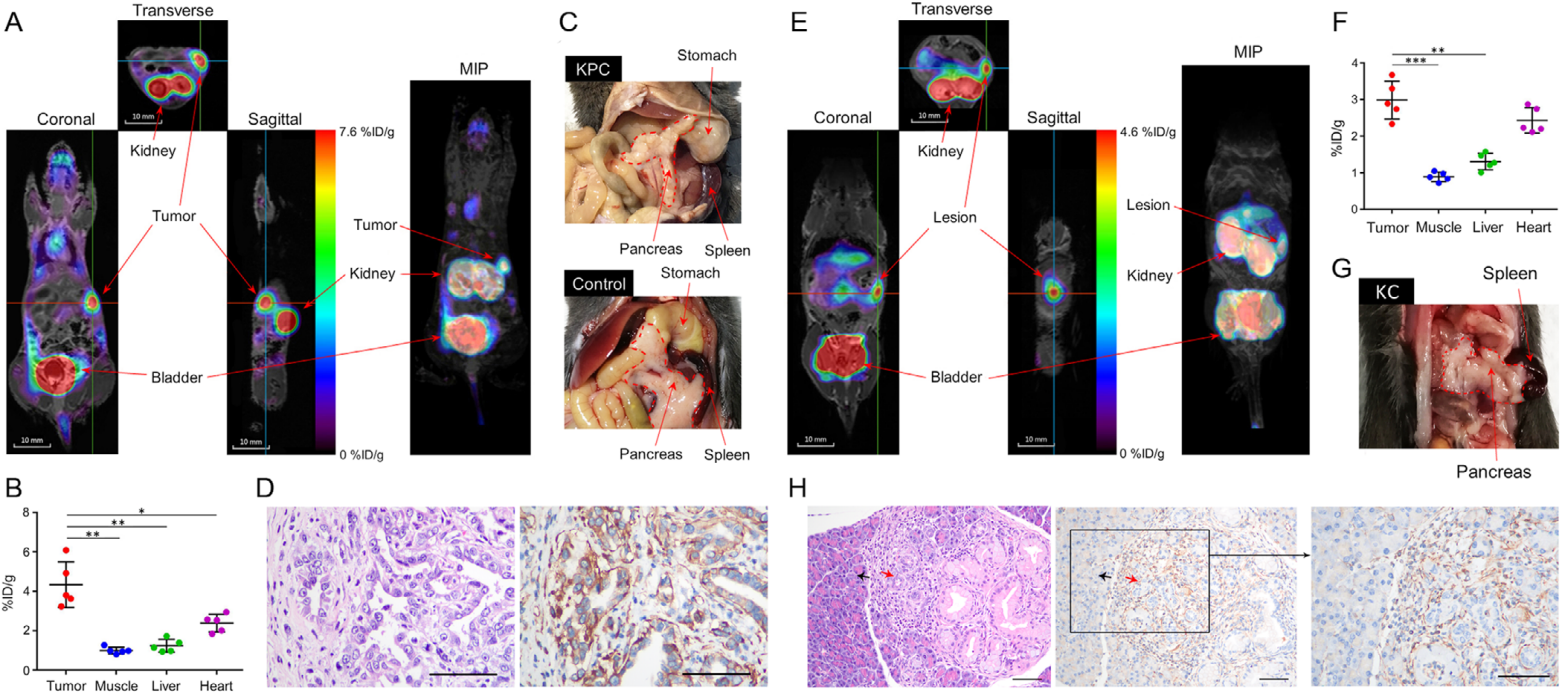
^18^F‐S5 PET/MR imaging in PDAC and PanIN mouse models. A, Representative cross‐sectional images of PET/MR imaging with ^18^F‐S5 of KPC mouse (left) and the 3D image (right). B, Quantification of ^18^F‐S5 uptake (%ID/g) by KPC mouse in tumor (4.34 ± 1.16), muscle (1.00 ± 0.17), liver (1.24 ± 0.33), and heart (2.38 ± 0.45), respectively. C, The pancreas of the KPC mouse showed an abnormal appearance compare to the pancreas of a healthy mouse. D, The development of PDAC in KPC mouse was confirmed by HE staining (left), and increased integrin α6 expression was confirmed by immunohistochemistry (right). E, Representative cross‐sectional images of PET/MR imaging of KC mouse (left) and the MIP reconstructed image (right). F, Quantification of ^18^F‐S5 uptake (%ID/g) by KC mouse in PanIN tissue (2.99 ± 0.52), muscle (0.89 ± 0.13), liver (1.31 ± 0.22), and heart (2.43 ± 0.35), respectively. G, The pancreas of the KPC mouse showed an abnormal appearance compare to the pancreas of a healthy mouse. H, The development of PanIN in mice was confirmed by HE staining (left), and the overexpression of integrin α6 in the lesion was analyzed using immunohistochemistry (right) (red arrow: PanIN; black arrow: relatively normal pancreatic tissue) (mean ± SD, the difference was evaluated by Student's *t*‐test, *P < .05, **P < .01, ***P < .001)

In summary, we developed an optimized integrin α6‐targeted radiolabeled probe, ^18^F‐S5, for PET/MR imaging of PDAC and PanIN in mice models. ^18^F‐S5 displayed ideal stability and biodistribution characteristics, and it enabled the detection of PDAC at an early stage with high sensitivity, a favorable tumor‐to‐muscle ratio, and low liver uptake in mice, which suggests its potential clinical translation.

## CONFLICT OF INTEREST

The authors have declared that no competing interest exists.

## ETHICS APPROVAL

The animal experiments were approved by the animal welfare and ethics committees of Sun Yat‐sen University Cancer Center.

## Supporting information

Supporting Information

## Data Availability

The raw data used in this article have been uploaded onto the Research Data Deposit public platform (www.researchdata.org.cn).
