# Supplementary material for: An optimized integrin α6‐targeted peptide for positron emission tomography/magnetic resonance imaging of pancreatic cancer and its precancerous lesion
Source: Clin Transl Med. 2020 Aug 26;10(4):e157. doi: 10.1002/ctm2.157 (PMC7449243; doi:10.1002/ctm2.157)

**Supplemental Material**

**Supplement Table 1. Causes of death (number of cases (%))**

|  | **ND**  **(N=24)** | **AD**  **(N=34)** | **VaD**  **(N=12)** | **AD/VaD**  **(N=21)** | **Pairwise p<0.05** |
| --- | --- | --- | --- | --- | --- |
| Dementia/  Neurologic cause | 0 (0%) | 23 (68%) | 1 (8%) | 9 (43%) | (ND) vs. (AD), (AD/VaD)  (AD) vs. (VaD) |
| Cardiovascular disease | 4 (17%) | 10 (29%) | 5 (42%) | 7 (33%) | None |
| Cancer | 16 (67%) | 2 (6%) | 2 (17% | 2 (10%) | (ND) vs. (AD), (VaD), (AD/VaD) |
| Respiratory disease | 2 (8%) | 2 (6%) | 2 (17%) | 2 (10%) | None |
| Natural cause | 0 (0%) | 0 (0%) | 2 (17%) | 3 (14%) | None |
| Failure to thrive | 2 (8%) | 0 (0%) | 0 (0%) | 0 (0%) | None |
| Trauma | 0 (0%) | 1 (3%) | 1 (8%) | 1 (5%) | None |

**Supplement Table 2. List of co-morbidities (number of cases (%))**

|  | **ND**  **(N=24)** | **AD**  **(N=34)** | **VaD**  **(N=12)** | **AD/VaD**  **(N=21)** | **Pairwise p<0.05** |
| --- | --- | --- | --- | --- | --- |
| Cardiovascular disease | 12 (50%) | 24 (71%) | 9 (75%) | 12 (57%) | None |
| Hypertension | 12 (50%) | 14 (41%) | 9 (75%) | 12 (57%) | None |
| Diabetes | 8 (33%) | 2 (6%) | 2 (17%) | 1 (5%) | (ND) vs. (AD), (AD/VaD) |
| Hyperlipidemia | 5 (21%) | 18 (53%) | 7 (58%) | 8 (38%) | (ND) vs. (AD) |
| Cancer | 16 (67%) | 5 (15%) | 4 (33%) | 7 (33%) | (ND) vs. (AD), (AD/VaD) |
| Respiratory disease | 6 (25%) | 5 (15%) | 3 (25%) | 5 (24%) | None |

**Supplement Table 3.** Correlogram showing correlation relationships (correlation coefficients) among cerebrovascular and neurologic pathologies.

|  | **Arteriole Medin** | **CoW atherosclerosis** | **CAA score** | **Tangle** | **Plaque** | **WML score** | **Expired age** |  |  |
| --- | --- | --- | --- | --- | --- | --- | --- | --- | --- |
| **Arteriole Medin** |  | 0.14 | 0.14 | 0.42 | 0.32 | 0.29 | 0.13 |  | **Legend** |
| **CoW atherosclerosis** | 0.14 |  | -0.11 | -0.02 | -0.10 | 0.27 | 0.59 |  | p<0.001 |
| **CAA score** | 0.14 | -0.11 |  | 0.5 | 0.55 | 0.25 | 0.08 |  | p<0.01 |
| **Tangle** | 0.42 | -0.02 | 0.5 |  | 0.80 | 0.42 | -0.05 |  | p<0.05 |
| **Plaque** | 0.32 | -0.10 | 0.55 | 0.80 |  | 0.24 | -0.10 |  |  |
| **WML score** | 0.29 | 0.27 | 0.25 | 0.42 | 0.24 |  | 0.38 |  |  |
| **Expired age** | 0.13 | 0.59 | 0.08 | -0.05 | -0.10 | 0.38 |  |  |  |

**Supplement Table 4**. Linear regression model to predict Total Tangle

| A. | Sample size | R^2^ adjusted | Multiple correlation coefficient |
| --- | --- | --- | --- |
| Model characteristics | 91 | 0.34 | 0.60 |
|  |  |  |  |
| Individual Variables | R partial | p-value |  |
| CAA score | 0.46 | <0.001 |  |
| Arteriole medin | 0.42 | <0.001 |  |

Variables not included in model: Circle of Willis atherosclerosis, expired age

| B. | Sample size | R^2^ adjusted | Multiple correlation coefficient |
| --- | --- | --- | --- |
| Model characteristics | 91 | 0.64 | 0.80 |
|  |  |  |  |
| Individual Variables | R partial | p-value |  |
| Total plaque | 0.75 | <0.001 |  |
| Arteriole medin | 0.22 | 0.04 |  |

Variables not included in model: CAA score, circle of Willis atherosclerosis, expired age

**Supplement Table 5**. Linear regression model to predict Total Plaque

|  | Sample size | R^2^ adjusted | Multiple correlation coefficient |
| --- | --- | --- | --- |
| Model characteristics | 91 | 0.36 | 0.60 |
|  |  |  |  |
| Individual Variables | R partial | p-value |  |
| CAA score | 0.49 | <0.001 |  |
| Arteriole medin | 0.38 | <0.001 |  |

Variables not included in the model: Circle of Willis atherosclerosis and expired age

**Supplement Table 6**. Linear regression model to predict CWML score

|  | Sample size | R^2^ adjusted | Multiple correlation coefficient |
| --- | --- | --- | --- |
| Model characteristics | 88 | 0.22 | 0.47 |
|  |  |  |  |
| Individual Variables | R partial | p-value |  |
| Expired age | 0.37 | <0.001 |  |
| Arteriole medin | 0.29 | 0.006 |  |

Variables not included in the model: Circle of Willis atherosclerosis and CAA score

Supplement Figure 1. **Cerebral arteriole medin and AD and VaD neuropathology**. A1 shows correlation between arteriole medin and total tangle score while A2 shows significantly higher medin with higher Braak stages. B1 shows correlation between arteriole medin and total plaque and B2 shows higher medin with higher plaque density score. C1 shows correlation between arteriole medin and white matter lesion score and C2 shows that donor brains with white matter lesions demonstrate higher arteriole medin.


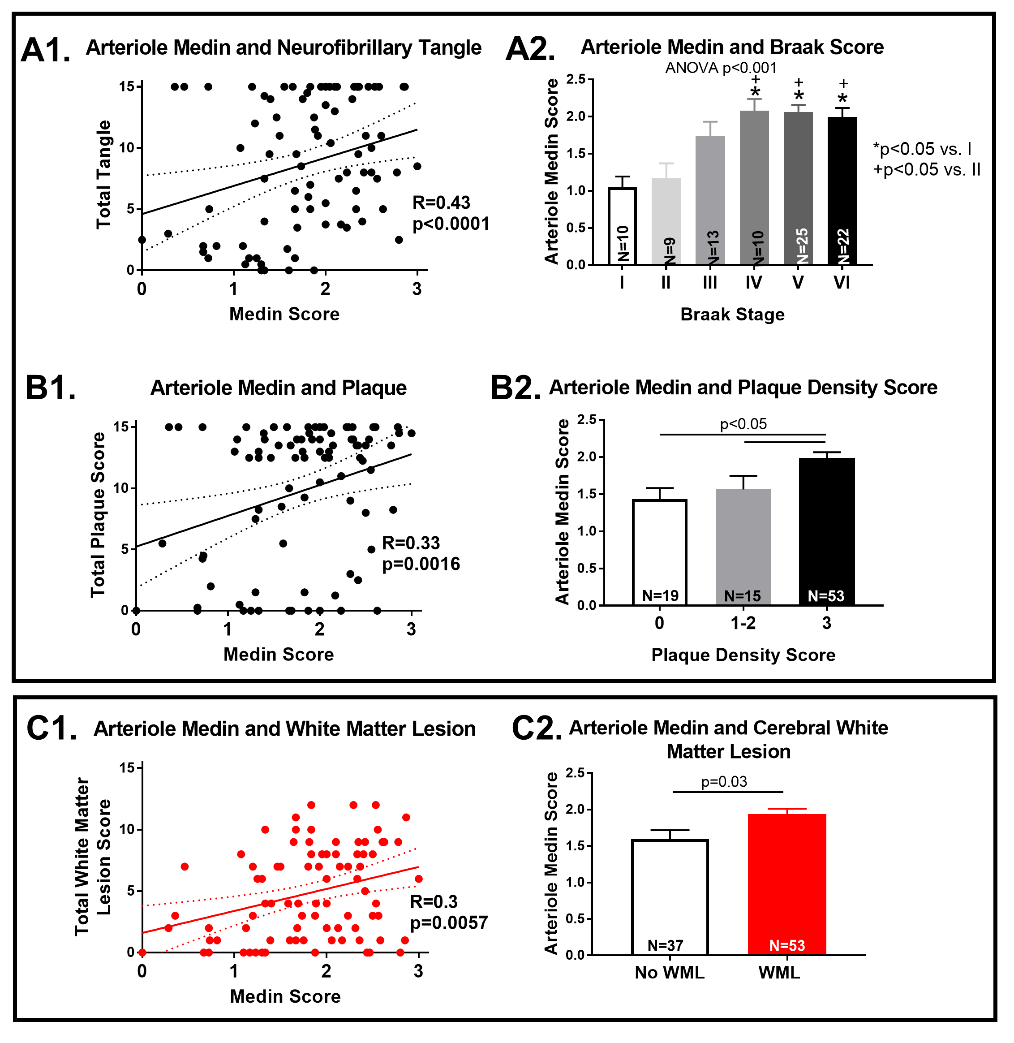

Supplement: Supplementary file 1 — Supporting Information [file CTM2-10-e157-s001.docx]
